# Gene Responses to Oxygen Availability in *Kluyveromyces lactis*: an Insight on the Evolution of the Oxygen-Responding System in Yeast

**DOI:** 10.1371/journal.pone.0007561

**Published:** 2009-10-26

**Authors:** Zi-An Fang, Guang-Hui Wang, Ai-Lian Chen, You-Fang Li, Jian-Ping Liu, Yu-Yang Li, Monique Bolotin-Fukuhara, Wei-Guo Bao

**Affiliations:** 1 Université Paris Sud-11, CNRS UMR 8621, Institut de Génétique et Microbiologie, Orsay, France; 2 School of Mathematics, Shandong University, Jinan, Shandong, China; 3 Department of Mathematics, Fuzhou University, Fuzhou, Fujian, China; 4 Institute of Genetics, State Key Laboratory of Genetic Engineering, School of Life Sciences, Fudan University, Shanghai, China; 5 Laboratoire Mathématiques Appliquées aux Systèmes, Ecole Centrale Paris, Châtenay-Malabry, France; University of Minnesota, United States of America

## Abstract

The whole-genome duplication (WGD) may provide a basis for the emergence of the very characteristic life style of *Saccharomyces cerevisiae*—its fermentation-oriented physiology and its capacity of growing in anaerobiosis. Indeed, we found an over-representation of oxygen-responding genes in the ohnologs of *S. cerevisiae*. Many of these duplicated genes are present as aerobic/hypoxic(anaerobic) pairs and form a specialized system responding to changing oxygen availability. *HYP2*/*ANB1* and *COX5A*/*COX5B* are such gene pairs, and their unique orthologs in the ‘non-WGD’ *Kluyveromyces lactis* genome behaved like the aerobic versions of *S. cerevisiae*. *ROX1* encodes a major oxygen-responding regulator in *S. cerevisiae*. The synteny, structural features and molecular function of putative *KlROX1* were shown to be different from that of *ROX1*. The transition from the *K. lactis*-type *ROX1* to the *S. cerevisiae*-type *ROX1* could link up with the development of anaerobes in the yeast evolution. Bioinformatics and stochastic analyses of the Rox1p-binding site (YYYATTGTTCTC) in the upstream sequences of the *S. cerevisiae* Rox1p-mediated genes and of the *K. lactis* orthologs also indicated that *K. lactis* lacks the specific gene system responding to oxygen limiting environment, which is present in the ‘post-WGD’ genome of *S. cerevisiae*. These data suggested that the oxygen-responding system was born for the specialized physiology of *S. cerevisiae*.

## Introduction

The yeast *Saccharomyces cerevisiae*, as well as other *sensu stricto Saccharomyces* species and a few of their close relatives, has a characteristic physiology that is obviously different from that of other eukaryotic cells and even most yeast species. One of the specialized features of *S. cerevisiae* is its regulatory network responding to oxygen availability, which allows this species to live on an exclusively fermentative mode even in fully aerated conditions, and also to grow vigorously in the complete absence of oxygen. Such a particular life style must rely on rapid and efficient gene responses to oxygen availability. The regulation of the oxygen-responding system, including both aerobic and hypoxic(anaerobic) genes that function either in presence or in absence of oxygen molecules, has been extensively investigated in *S. cerevisiae*, and the major transcription activators and repressors such as Hap1p, Rox1p and Mot3p have been identified and characterized [1]–[6].

It has been proposed that a whole-genome duplication (WGD) event occurred after the separation of *S. cerevisiae* and *Kluyveromyces lactis* from a common ancestral ‘pre-WGD’ yeast [7]–[9]. Some recent analyses suggested that the WGD could provide the basis for the evolution that led to the special physiological properties of the modern *S. cerevisiae* yeast containing a ‘post-WGD’ genome [10], [11]. For example, an increase in the number of some genes resulted in an enhanced glycolytic flux, which is necessary for the fermentation-oriented metabolism of *S. cerevisiae* [12]. This change was regarded as an outcome of the WGD event followed by a selection in glucose-rich environments [13].

Among massive duplicated genes of *S. cerevisiae* (most of which were formed by whole-genome duplication, and called ohnologs-a subset of paralogs), many gene pairs were combined into a network system specially involved in oxygen response. *HYP2*/*ANB1* and *COX5A*/*COX5B* are thought to be such gene pairs. While *HYP2* and *COX5A* work in aerobic condition, *ANB1* and *COX5B* function in hypoxic and anaerobic conditions [14]–[16]. This oxygen-dependent alternative expression pattern of aerobic versus hypoxic(anaerobic) genes can also be found in many other cases of the duplicated gene pairs, such as *CYC1*/*CYC7* [17], *PET9*/*AAC3* [18], and *HMG1*/*HMG2* [19]. A trait shared with these gene pairs is that their hypoxic(anaerobic) partners are commonly under the repression of Rox1p in an oxygen-dependent way [6]. Some hypoxic(anaerobic) genes such as *ANB1* and *HEM13* are also regulated by another repressor Mot3p [2], [20]–[22].

As opposed to *S. cerevisiae*, *K. lactis* shows a quite different oxygen response. It is Crabtree effect-negative [10] and Kluyver effect-positive [23], like many other yeast species, whilst *S. cerevisiae* is not. Further, *K. lactis* contains a ‘non-WGD’ genome [24] in which there are sets of genes comparable to those of *S. cerevisiae* [25]. The genomic and physiological features of *K. lactis* suggest that this yeast may have retained some major characteristics of the oxygen response patterns of the ancestral genome that gave rise to *S. cerevisiae* and *K. lactis*. The regulation of a few genes involved in respiration and haem synthesis in *K. lactis* has been reported to be dependent on oxygen [26]–[28]. Our previous work revealed that the target genes and regulatory modes of the oxygen-dependent regulator *Kl*Hap1p in *K. lactis* were different from those of the *S. cerevisiae* Hap1p [29]. However, the information is still limited about the gene response to oxygen availability and, in particular, about its regulators in *K. lactis*. We therefore selected some *K. lactis* genes, which appeared equivalent to the well-known oxygen-responding genes in *S. cerevisiae* such as *HEM13*, *HYP2*/*ANB1* and *COX5A*/*COX5B*, and investigated the gene response to oxygen in *K. lactis*, in an attempt to understand how the oxygen-responding system has evolved from a ‘pre-WGD’ to a ‘post-WGD’ genome.

## Results

### Oxygen-responding genes are over-represented in ohnologs of *S. cerevisiae*

**Figure 1:**
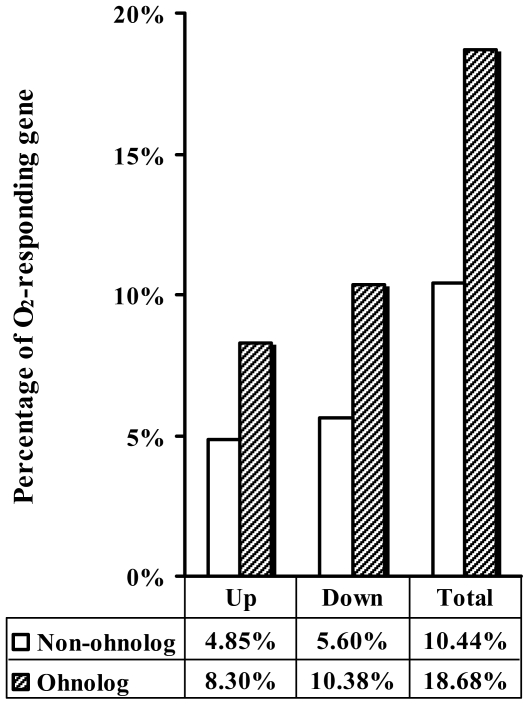
Distribution of oxygen-responding genes in *S. cerevisiae*. Genes are counted that showed more than 2-fold change of expression level (up- or down-regulation) in response to oxygen. Hatched and blank boxes represent the percentages of oxygen-responding genes in onhologs and non-onhologs respectively.

It has been reported that hundreds of genes respond to oxygen in *S. cerevisiae* [30]. In this ‘post-WGD’ genome, 554 ohnolog pairs have been identified (http://wolfe.gen.tcd.ie/ygob/) [9], [31]–[33]. To estimate the role of the ohnologs in oxygen response, we checked the expression of these genes under aerobic and anaerobic conditions according to the published microarray survey including 6020 ORFs [30] (also see http://transcriptome.ens.fr/ymgv/publi_desc.php?pub_id=34). The results are included in **Supplementary Table S1**. With respect to a factor of 1.5, 2 or 3 in the change of the gene expression level between aerobic and anaerobic conditions, a significantly higher proportion of genes responding to oxygen were always found in ohnologs than the genes scattered over the whole genome. This is the case for both up- and down- regulated genes **(Supplementary Table S2-A)**. The results indicated that oxygen-responding genes arose from ohnologs more frequently than from non-ohnologs (**Supplementary Table S2-B**) in the evolution of the *S. cerevisiae* genome. For example, considering a factor of 2, 18.7% of ohnologs were oxygen responsive, but only 10.4% in non-ohnologs (**Figure 1**). The former is 78.9% higher than the latter (*P*<e−15). There are 174 ohnolog pairs among 554, in which at least one gene has oxygen response, indicating that nearly one third of genes (31.4%) became involve in response to oxygen availability after the WGD event. These data suggested a connection between the formation of efficient oxygen-responding system in *S. cerevisiae* and the whole-genome duplication event, which has been previously discussed [34], [35].

### Aerobic/hypoxic(anaerobic) gene pairs in *S. cerevisiae* usually appear unique in *K. lactis*

There are many aerobic/hypoxic(anaerobic) paralog pairs in *S. cerevisiae*, such as *HYP2*/*ANB1* and *COX5A*/*COX5B* [3], [30], [36]. Either gene in each pair functions differently when environmental oxygen concentration changes, constituting an optimized oxygen response network. Most of these paralogs are in the list of ohnolog pairs, and might be formed as an outcome of selection for oxygen response after the whole-genome duplication. There are also a few exceptions among the aerobic/hypoxic(anaerobic) paralog pairs. For example, *OYE3*/*OYE2* [37] and *AAC1*/*AAC3* (a triplet including *PET9* (*AAC2*)) [18] could derive from individual gene duplication. Sequence similarity search revealed that such aerobic/hypoxic(anaerobic) pair mostly has only one ortholog in *K. lactis* (**Table 1**). We may ask how these singular genes behave to respond to oxygen availability in *K. lactis* and question whether *K. lactis* and *S. cerevisiae* share similar gene categories involved in oxygen response.

**Table 1:** Some examples of aerobic/hypoxic(anaerobic) paralog pairs in *S. cerevisiae* and their orthologs in *K. lactis*.

Function (annotation in *S. cerevisiae*)	*S. cerevisiae* *K. lactis*
	Gene (ORF)	ORF	Score bits	(E value) *
cytochrome c	*CYC1* a	KLLA0F16940g	202	(7e−54)
	*CYC7* ^§^		188	(1e−49)
translation initiation factor eIF-5A	*HYP2* a	KLLA0E22286g	281	(3e−77)
	*ANB1* ^§^		285	(2e−78)
cytochrome c oxidase chain V	*COX5A* a	KLLA0F03641g	221	(4e−59)
	*COX5B* ^§^		200	(6e−53)
mitochondrial inner membrane ADP/ATP translocator	*AAC1* a	KLLA0E12353g	429	(e−121)
	*PET9* (*AAC2*)^a^		513	(e−146)
	*AAC3* ^§^		473	(e−134)
3-hydroxy-3-methylglutaryl-coenzyme A (HMG-CoA) reductase	*HMG1* a	KLLA0B04642g	1250	(0.0)
	*HMG2* ^§^		1062	(0.0)
mitochondrial matrix protein, scaffold of iron-sulfur cluster assembly	*ISU1* a	KLLA0D07161g	233	(9e−63)
	*ISU2* ^§^		234	(3e−63)
ceramide synthase component	*LAG1* a	KLLA0B13497g	482	(e−137)
	*LAC1* ^§^		532	(e−152)
suppressor of DNA polymerase mutations	*PSP1* a	KLLA0C01716g	427	(e−120)
	*YLR177W* ^§^		454	(e−128)
protein disulfide isomerase	*PDI1* a	KLLA0C01111g	500	(e−142)
	*EUG1* ^§^		371	(e−103)
phosphoglucomutase	*PGM1* a	KLLA0B12694g	797	(0.0)
	*PGM2* ^§^		822	(0.0)
mitochondrial porin (voltage-dependent anion channel)	*POR1* a	KLLA0F03553g	369	(e−103)
	*POR2* ^§^		280	(2e−76)
transcription factor of sterol biosynthesis or sterol uptake	*ECM22* a	KLLA0A04169g	423	(e−119)
	*UPC2* ^§^		552	(e−158)
acyl-CoA:sterol acyltransferase	*ARE2* a	KLLA0C09152g	516	(e−147)
	*ARE1* ^§^		478	(e−136)
NADPH oxidoreductase containing flavin mononucleotide (FMN)	*OYE3* a	KLLA0A09075g	563	(e−161)
	*OYE2* ^§^		607	(e−175)

* : BLAST search was carried out at Génolevures http://cbi.labri.fr/Genolevures/.

a : aerobic paralog; ^§^: hypoxic(anaerobic) paralog in *S. cerevisiae*.

### *KlHEM13*, *KlHYP2*(*ANB1*) and *KlCOX5A*(*5B*) show differential responses to oxygen availability in *K. lactis*

**Figure 2:**
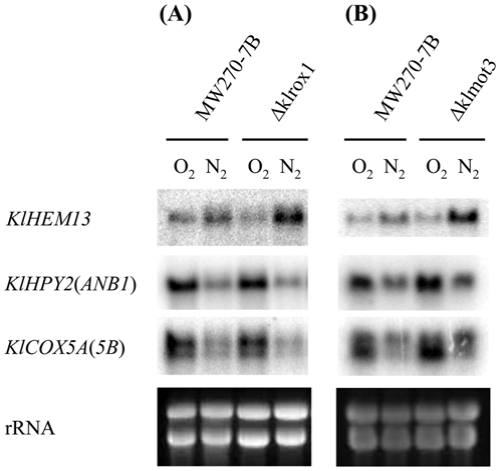
Response of *KlHEM13*, *KlHPY2*(*ANB1*) and *KlCOX5A*(*5B*) to oxygen availability in *K. lactis*. Cells were grown to OD_600_ ∼4 at 28°C in complete YP glucose medium supplemented with ergosterol and Tween 80 under aerobic (O_2_) condition. A half of the culture was used for hypoxic treatment (N_2_) as described in the “Materials and Methods”, and then incubated at 28°C for a further 6 hours. Total RNA was extracted and Northern hybridization was performed to probe *KlHEM13*, *KlHYP2*(*ANB1*) and *KlCOX5A*(*5B*) transcripts (see Materials and Methods). Panel A: the *K. lactis* wild type MW270-7B and its isogenic mutant Δ*klrox1*. Panel B: MW270-7B and its isogenic mutant Δ*klmot3*. The RNA samples of MW270-7B in panels A and B were independent preparations. The rRNA was used for quantifying sample loads.

*HEM13*, *HYP2/ANB1* and *COX5A/COX5B* are well-known genes responding to oxygen in *S. cerevisiae* [14], [16], [20], [38]–[42]. These genes play important roles in heme synthesis, translational initiation and mitochondrial respiratory chain biogenesis, respectively. We therefore investigated the expression of their *K. lactis* orthologs under both aerobic condition and during the shift to hypoxic condition. The results of Northern hybridization are shown in **Figure 2A and 2B**.

*HEM13* of *S. cerevisiae* encodes coproporphyrinogen III oxidase [43]. *KlHEM13*, its counterpart in *K. lactis*, is a singleton gene. The transcript level was slightly increased under hypoxic condition, though not as much as that of *HEM13* in *S. cerevisiae*. This response of *KlHEM13* was the same as that reported previously [29], in which cells were grown under hypoxic condition instead of here a shift from aerobic to hypoxic conditions.

Both *HYP2/ANB1* and *COX5A/COX5B*, encoding translation initiation factor elF-5A [44] and subunit V of cytochrome c oxidase [45] respectively, are ohnolog pairs in *S. cerevisiae* [9], [31]–[33]. Each of these two gene pairs has only one unique ortholog, *KlHYP2*(*ANB1*) and *KlCOX5A*(*5B*) respectively, in the *K. lactis* genome. Analyses of sequence similarity (**Table 1**) and genomic context (http://wolfe.gen.tcd.ie/cgi/browser/ygob.pl?gene), however, could not decide which gene in the pair was closest to the *K. lactis* ortholog. For example, *KlCOX5A*(*5B*) shows more similarity to *COX5A* in sequence, but is more related to *COX5B* in terms of synteny and by presence of an intron. The situation is the same for *KlHYP2*(*ANB1*). Northern hybridization showed that the transcription of both *KlHYP2*(*ANB1*) and *KlCOX5A*(*5B*) was markedly reduced under hypoxic condition (**Figure 2A and 2B**), very much like the aerobic genes *HYP2* and *COX5A* in the *S. cerevisiae* pairs, but unlike the hypoxic(anaerobic) *ANB1* and *COX5B*. It is worth noting that *KlCOX5A*(*5B*) showed two transcripts of different size. The meaning of this observation is unknown, but a similar phenomenon was also reported for *KlCYC1* [46], [47] and *HGT1* [48].

Simultaneous deletion of both *HYP2* and *ANB1* is lethal for *S. cerevisiae* [15]. Therefore the singular *KlHYP2*(*ANB1*) could be also necessary for viability of *K. lactis*. In this sense, the decreased expression of *KlHYP2*(*ANB1*) under oxygen limited conditions in *K. lactis* may be a reason for its inability to grow in anaerobiosis. The *cox5acox5b* double deletion mutants of *S. cerevisiae* are completely non-respiratory and only grow by fermentation [49]. Inactivation of respiration in *K. lactis* (for example disruption of the single cytochrome c gene *KlCYC1*) leads to severe growth defect even on glucose because of its limited fermentation capacity [50]. Thus, *KlCOX5A*(*5B*) must be important for growth of *K. lactis*, even under aerobic condition. Therefore, the growth impact of a reduced *KlCOX5A*(*5B*) expression under hypoxic condition could be another possible reason of the distinct oxygen response of *K. lactis*.

### Analysis of putative *ROX1* orthologs suggests that there was a transition from the *K. lactis*-type *ROX1* to the *S. cerevisiae*-type *ROX1*

**Figure 3:**
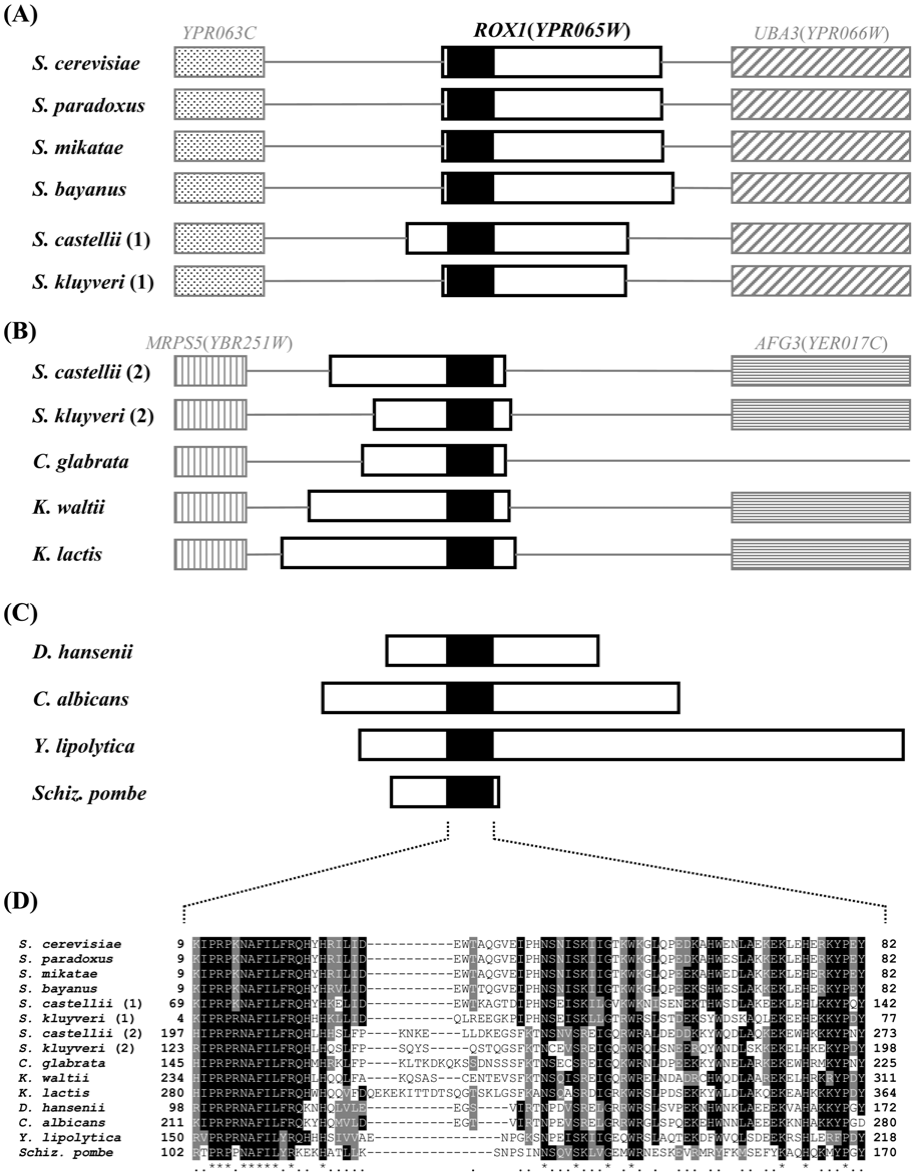
Conservation and divergence of the *ROX1*-like HMG-encoding genes in yeasts. Panels A, B and C: the blank box indicates the HMG-encoding gene in which the solid box shows the location of the HMG-domain. The sizes are in proportion. Other types of boxes represent the chromosomal neighbors of *ROX1* or *KlROX1* and their orthologs in different yeast species. Panel D: alignment of the HMG-domains from various yeasts. The numbers indicate the amino-acid positions in the proteins. The asterisk and dot below the alignment show identity or conservative replacement of amino acid, respectively.

*ROX1* encodes a major repressor of the expression of many oxygen-responding genes such as *HEM13*, *ANB1* and *COX5B* in *S. cerevisiae* [6], [51]. The differential oxygen responses of *KlHEM13*, *KlHYP2*(*ANB1*) and *KlCOX5A*(*5B*) led us to question whether there exists a *ROX1* ortholog in *K. lactis*. By a BLAST search using the amino acid sequence of Rox1p, we found in the *K. lactis* genome (Genolevures: http://www.genolevures.org/) a possible equivalent of *ROX1*: KLLA0B11495g, but with a limited E-value (7e−13). We designated the gene *KlROX1*. It predicts a protein of 393 amino acids, 45-kDa, in which a HMG (high-mobility group) domain was well conserved (44% identical) while other parts have diverged from Rox1p (368 amino acids). The HMG domain is localized at C-terminal part of *Kl*Rox1p, while it is at N-terminal part of Rox1p (**Figure 3A and 3B**). Also, there is no syntenic relation for these two genes. *YPR063C* and *UBA3*(*YPR066W*) are at upstream and downstream of *ROX1* in *S. cerevisiae*, whilst the neighbour genes for *KlROX1* are orthologous to *MRPS5*(*YBR251W*) and *AFG3*(*YER017C*) respectively (**Figure 3A and 3B**).

As described above, we defined *KlROX1* solely on the basis of BLAST search. Its sequence similarity to *ROX1* is limited. We may ask: do there exist such two kinds of *ROX1* orthologs among yeasts? To examine this possibility, we analyzed putative *ROX1* orthologs in other yeast species. In the yeasts *S. paradoxus*, *S. mikatae* and *S. bayanus* which are closely related to *S. cerevisiae*, a sequence significantly similar to the *S. cerevisiae ROX1* (E value <e−88) was found in each of these genomes. The contexts of upstream *YPR063C* and downstream *UBA3*(*YPR066W*) genes are also conserved in these three yeasts (**Figure 3A**). Although BLAST results showed that the similarity was restricted to the HMG part in the Rox1p orthologs of *S. castellii* (1.5e−27) and *S. kluyveri* (5e−32), some sequence features such as glutamine stretches are still found in these proteins. Analysis of HMG localization and synteny indicated that these two genes are likely orthologs of the *S. cerevisiae ROX1* (**Figure 3A**). However, these two yeasts contain another gene that may be orthologous to *KlROX1* (4.1e−15 and 7e−19) (**Figure 3B**). The *K. lactis*-type gene, instead of the *S. cerevisiae*-type gene, is also present in the genomes of *C. glabrata* (2e−20) and *K. waltii* (3e−15) (**Figure 3B**). In the taxonomically distant yeast species, *D. hansenii* (9e−24), *C. albicans* (1.7e−20), *Y. lipolytica* (5e−18) and *Schiz. pombe* (3.9e−13), there still exist genes encoding HMG-containing proteins. However, except for a conserved HMG domain (**Figure 3D**), these genes differ much in their structural and syntenic features (**Figure 3C**).

Thus, in the process of the yeast evolution from the ancestral ‘pre-WGD’ species to the ‘post-WGD’ species, the HMG-encoding genes could have undergone a development from the *K. lactis*-type *ROX1* gene to the *S. cerevisiae*-type *ROX1* gene, through an intermediary co-existence of *KlROX1* and *ROX1*. Importantly, we found that the formation of the *S. cerevisiae*-type *ROX1* gene could connect with the development of the capability of anaerobic growth in the yeast evolution, as shown in **Table 2**.

**Table 2:** Correlation of anaerobic growth with the presence of the *S. cerevisiae*-type *ROX1* in the evolution of yeast.

Species	Anaerobic growth	Putative *ROX1*		Allantoin degradation			DHODase		Salvage NAD synthesis
		*Sc*-type	*Kl*-type	*DAL* cluster	*UAP*	*UOX*	cytoplasmic	mitochondrial	
S. cerevisiae	*+*	+	−	+	−	−	+	−	+
*sensu stricto*	*+*	+	−	+	−	−	+	−	+
S. castellii	*+*	+	+	+	−	−	+	−	+
S. kluyveri	*+*	+	+	−	+	+	+	+	+
C. glabrata	*−*	−	+	−	−	−	−	+	+
K. waltii	*−*	−	+	−	+	+	+	+	+
K. lactis	*−*	−	+	−	+	+	+	+	+
C. albicans	*−*	−	−	−	+	+	−	+	+
Y. lipolytica	*−*	−	−	−	+	+	−	+	+
Schiz. pombe	*−*	−	−	−	+	+	−	+	+

The line *sensu stricto* refers to *S. paradoxous*, *S. mikatae*, *S. bayanus*.

Sequences as queries (NCBI accession numbers): UAP (CAA50681), UOX (P78609), DHODases (NP_012706 and NP_593317).

### Oxygen-dependent regulation of *KlHEM13*, *KlHYP2*(*ANB1*) and *KlCOX5A*(*5B*) is not mediated by *KlROX1* in *K. lactis*

The analysis of possible *ROX1* orthologs found in various yeast species has raised the question whether the changes from the *K. lactis*-type *ROX1* to the *S. cerevisiae*-type *ROX1* accompany a functional alteration of these HMG-encoding genes. In order to see whether *KlROX1* has a transcriptional repression function similar to that of the *S. cerevisiae ROX1*, a Δ*klrox1* null mutant was constructed and used in the investigation of oxygen response in *K. lactis*. Northern hybridization showed that the *klrox1* mutation had no obvious effect in the expression of *KlHEM13*, *KlHYP2*(*ANB1*) and *KlCOX5A*(*5B*), neither in aerobic nor hypoxic conditions (**Figure 2A**). Therefore, *KlROX1* is not involved in the transcriptional repression of these three *K. lactis* genes that are orthologous to the well-known oxygen-responding genes of *S. cerevisiae*.

### *Kl*Rox1p is not functionally equivalent to Rox1p in the repression of *HEM13*

**Figure 4:**
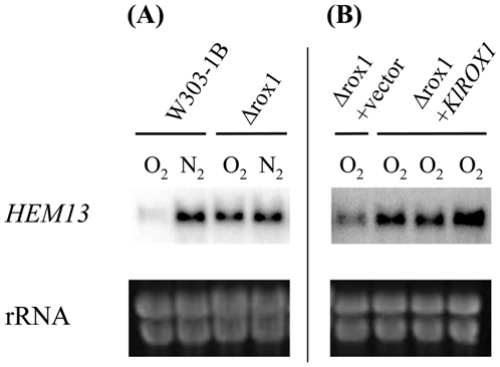
Difference between Rox1p and *Kl*Rox1p in the repression on *HEM13* of *S. cerevisiae*. Cells were grown to OD_600_ 1.5 to 2.0 at 28°C in minimal glucose medium supplemented with ergosterol and Tween 80 under aerobic (O_2_) or hypoxic (N_2_) conditions. Total RNA was extracted and Northern hybridization was performed to probe *HEM13* transcript (see Materials and Methods). Panel A: the *S. cerevisiae* wild type W303-1B and its isogenic mutant Δ*rox1*. Panel B: the Δ*rox1* mutant transformed with the empty vector pCM262 and the plasmid carrying *KlROX1* in which three independent *KlROX1* clones were included. The rRNA was used for sample loading quantification.

In *S. cerevisiae*, the repression of Rox1p on the *HEM13* expression has been extensively investigated [6], [38], [51]. Exactly as reported, deletion of *ROX1* derepressed the expression of *HEM13* under aerobic growth (**Figure 4A**). The *KlROX1* gene on a 2μ-based vector was then transformed into the *S. cerevisiae* Δ*rox1* mutant, and expression of *Kl*Rox1p was confirmed by Western blot (data not shown). As compared to the empty vector, introduction of *KlROX1* led to an activation rather than to a repression on *HEM13* expression (**Figure 4B**), suggesting different molecular functions between *Kl*Rox1p and Rox1p.

### Search for the Rox1p-binding site YYYATTGTTCTC in the upstream regions of the *K. lactis* genes orthologous to the *S. cerevisiae* Rox1p target genes

In *S. cerevisiae*, Rox1p controls its target genes through binding to *cis*-acting elements with consensus sequence YYYATTGTTCTC [6]. The HMG domain is responsible for the protein-DNA interaction [52], [53] and its binding induces a topological change of 90° bending of DNA [54]. Up to now, other genes coding for HMG-containing proteins (**Figure 3C**) have been characterized in *Schiz. pombe* [55], [56] and *C. albicans* [57]–[59]. They encode either a mating-type M-specific polypeptide or a repressor of filamentous growth, the functions of which are completely divergent from that of Rox1p in *S. cerevisiae*. Although not involved in oxygen response, both proteins of *Schiz. pombe* and *C. albicans* could bind specifically to the YYYATTGTTCTC site, and the binding was dependent on the HMG domain [56], [59], [60].

**Figure 5:**
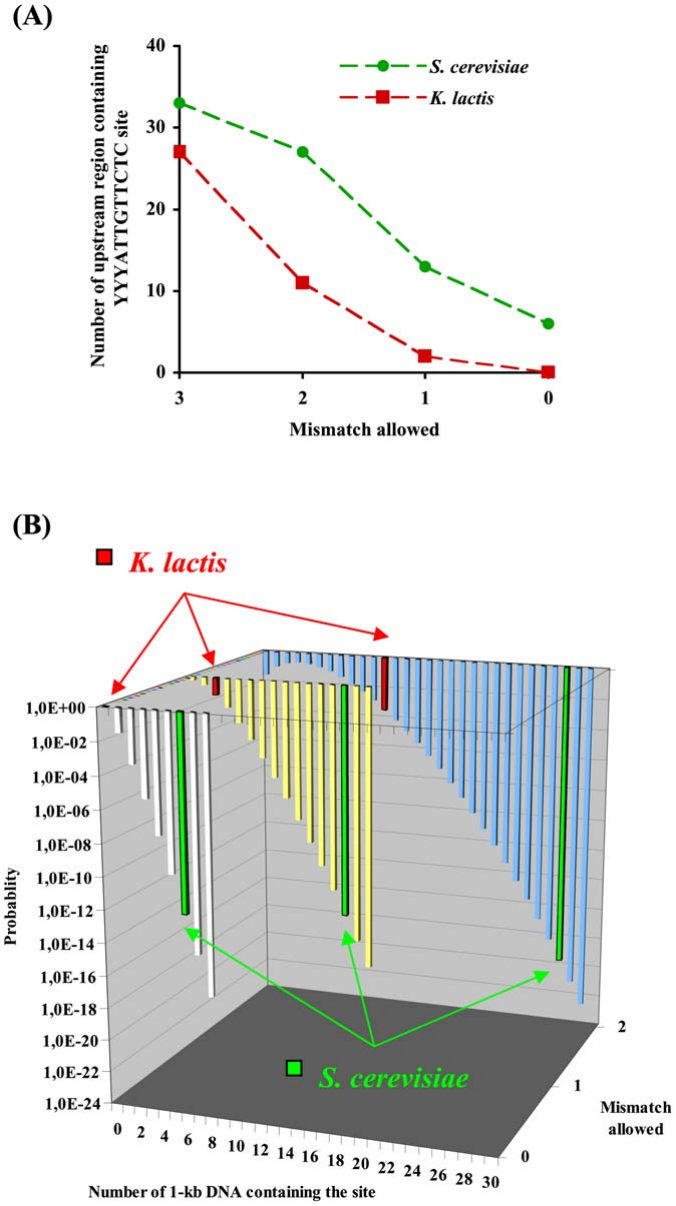
Occurrence of the Rox1p-binding site. Panel A: Comparison of the upstream regions (1 kb) containing the site YYYATTGTTCTC between the Rox1p-mediated hypoxic genes of *S. cerevisiae* (green circle) and their orthologs of *K. lactis* (red square). Panel B: stochastic model about the presence of the YYYATTGTTCTC site in a system containing 37 1-kb DNA sequences. Series white, prefect match; series yellow, 1 mismatch allowed; series blue, 2 mismatches allowed. Green bars represent the probabilities corresponding to the situations in *S. cerevisiae* and red in *K. lactis*.

Since the HMG domain is well conserved in *Kl*Rox1p (**Figure 3D**), we may expect that it would possess some of the Rox1p functions. *Kl*Rox1p could activate, but not repress, the expression of *HEM13* in the *S. cerevisiae* Δ*rox1* mutant (**Figure 4**). We therefore supposed that the *K. lactis* protein, like the HMG proteins of other yeast species, could bind the YYYATTGTTCTC site. We then investigated the possible site in the upstream regions of the *K. lactis* genes, which are orthologous to the *S. cerevisiae* oxygen-responding genes mediated by Rox1p according to the data previously reported [3], [4], [36], [51]. While *DAN1*, *YHK8*, *TIR2*, *YGR035C*, *YAR028W* and *IRC23* are absent, 37 genes have their orthologs in *K. lactis* as revealed by BLAST search, in which 4 genes may have 2 putative *K. lactis* orthologs (**Supplementary Table S3**). 7 sites perfectly matched with the YYYATTGTTCTC motif present in 6 promoters of the *S. cerevisiae* genes, but no such site was found in any upstream sequences of the *K. lactis* genes. When 1 mismatch was allowed, only 2 upstream regions were detected 1 site each in *K. lactis*, much less than that in *S. cerevisiae* (13 promoters containing total 15 sites). The situation was similar when 2 mismatches were allowed (*K. lactis*: 13 sites in 11 upstream regions, *S. cerevisiae*: 43 in 27) (**Supplementary Table S3** and **Figure 5A**).

### *KlMOT3* is dispensable for the regulation of the *KlHEM13*, *KlHYP2*(*ANB1*) and *KlCOX5A*(*5B*) in *K. lactis*

Our data above suggested that the Rox1p-mediated oxygen-responding system might not exist in *K. lactis*. Besides *ROX1*, *MOT3* has also been reported to be involved in repressing a subset of hypoxic genes such as *HEM13* and *ANB1* in *S. cerevisiae* [20]–[22], [51]. By BLAST search using the amino-acid sequence of the *S. cerevisiae* Mot3p, we found a candidate ortholog KLLA0E18645g (designated *KlMOT3*) in *K. lactis*, with a moderate E-value (7.6e−28). The similarity between *Kl*Mot3p and Mot3p is concentrated in the C-terminal part containing two Cys2-His2 zinc fingers. But the gene synteny is conserved among different yeast species (http://wolfe.gen.tcd.ie/cgi/browser/ygob.pl?gene=YMR070W). To address whether *K. lactis* contains a functional *MOT3* ortholog or not, a Δ*klmot3* null mutant was constructed to investigate its possible role in oxygen response of *K. lactis*. Northern hybridization indicated that the expression of *KlHEM13*, *KlHYP2*(*ANB1*) and *KlCOX5A*(*5B*) was not affected in the Δ*klmot3* mutant, under either aerobic or hypoxic conditions (**Figure 2B**). The result suggested that *KlMOT3*, as well as *KlROX1*, is not involved in the transcriptional repression of these three *K. lactis* genes expression and that *KlMOT3* and *MOT3* are not functional equivalents in their role of oxygen response.

## Discussion

### The pre-WGD genome of *K. lactis* might lack a specific gene system responding to oxygen-limiting environment

In *S. cerevisiae*, the transcriptional repressor Rox1p and its target genes such as *ANB1* and *COX5B* form a network devoted to respond to low oxygen environments. This repressor seems absent in *K. lactis* and the genes orthologous to *ANB1* and *COX5B* showed a down-regulation response to low oxygen condition. Our results suggested that the Rox1p-mediated response to oxygen is not operating in this non-WGD yeast species. This proposition is consistent with a recent observation in which the hypoxic *AAC3* gene of *S. cerevisiae* was expressed constitutively in *K. lactis* under both aerobic and hypoxic conditions [28].

The upstream YYYATTGTTCTC site connects the Rox1p target genes to the oxygen response in *S. cerevisiae*. We found that this site is much less abundant in the upstream regions of the forty-one *K. lactis* genes orthologous to the thirty-seven Rox1p target genes of *S. cerevisiae*. To evaluate the significance of such difference in the site occurrence, we calculated the stochastic probabilities that the YYYATTGTTCTC site is present at 0, 1, 2, 3, ……, n 1-kb DNA sequences in a system of thirty-seven members (**Figure 5B**) (the result was similar for a system of forty-one members). There was not any the perfectly matched site in the upstream regions of the *K. lactis* genes, the corresponding probability in the stochastic system was 0.97-almost a certain event. In the cases of 1 and 2 mismatches allowed, the probabilities were 0.08 and 3.17e−4 for *K. lactis*. On the contrary, all the probabilities for *S. cerevisiae* were 1.59e−12, 3.33e−15 and 2.11e−20 respectively. These data indicated that the *K. lactis* system is almost (or close to) a random site distribution while the *S. cerevisiae* system is highly selected with respect to the YYYATTGTTCTC site, suggesting that these *K. lactis* genes have not associated into a regulatory network which can respond synergistically to oxygen availability.

### Appearance of the *S. cerevisiae*-type *ROX1* gene could be a hallmark that links the ability of anaerobic growth in some yeasts

Some metabolites can be produced through either oxygen-dependent pathway or oxygen-independent pathway. Utilization of oxygen independent or less-dependent pathway is the reasonable choice for growth under oxygen limiting conditions. For example, NAD is synthesized from both tryptophan and niacin by the oxygen-requiring kynurenine pathway and the alternative oxygen-independent salvage pathway in *S. cerevisiae*. The salvage pathway has been proved to be necessary for the anaerobic growth for *S. cerevisiae* [61], but the variation in the modes of NAD acquisition in yeasts seems to have no direct link to the ability of anaerobic growth [62], since the salvage pathway for NAD synthesis exists in all yeast species including strict aerobes such as *Y. lipolytica* and *Schiz. pombe* (**Table 2**). However, it has been found that some metabolic processes are reconfigured to avoid dependence on oxygen-requiring reactions or respiration in the evolution towards anaerobic or hypoxic growth. For example, the enzyme dihydroorotate dehydrogenase (DHODase) in pyrimidine synthesis pathway was converted from a mitochondrial component into a cytoplasmic protein by horizontal gene transfer [63]–[66]. Also, birth of *DAL* gene cluster and loss of genes encoding urate oxidase (UOX) and urate permease (UAP) led a switch from urate to allantoin utilization [67]. These biochemical reorganizations in the yeast evolution would economize oxygen consummation to adapt the life under oxygen-poor or –depleted environments.

Our analyses indicated that the *S. cerevisiae*-type *ROX1* is specific for anaerobes including *S. castellii* and *S. kluyveri* [64], [65], [68] and it is absent in aerobes (**Table 2**). *ROX1* and its target hypoxic genes form a specialized network that actively takes challenges of oxygen-limiting or -absent conditions.

### Formation of the Rox1p-mediated hypoxic genes and whole-genome duplication

**Figure 6:**
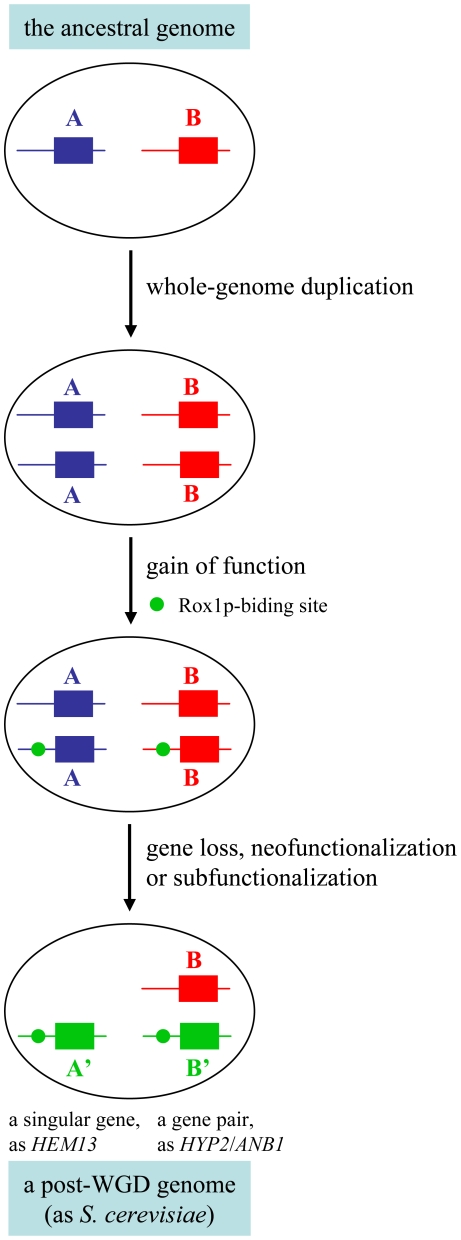
Cartoon illustration of a model about the establishment of the Rox1p-mediated O_2_-reponding genes in the post-WGD genomes. A and B represent genes that existed in the ancestral genome, A′ and B′ are modified versions of A and B after the whole-genome duplication.

The transcription analyses in this work revealed the expression of some important genes for cell viability such as *KlHYP2*(*ANB1*) and *KlCOX5A*(*5B*) was significantly repressed by hypoxic condition in the pre-WGD species *K. lactis*. Yeast has to devise a gene version capable of hypoxic(anaerobic) expression in order to survive in the absence of oxygen. Among 37 targets of Rox1p, 16 (43.2%) genes have an ohnolog and 13 (35.1%) singular genes are located within the duplication blocks (**Supplementary Table S3**), suggesting that as high as 78% of Rox1p-mediated genes may originate from the whole-genome duplication event. The whole-genome duplication would provide a basis for the construction of a hypoxic(anaerobic) working system with many genes. After the duplication, acquisition or creation of Rox1p-binding site could render one gene of a duplicated pair to become a hypoxic(anaerobic) version (**Figure 6**). Ideally, the paired genes function concertedly under aerobic and hypoxic(anaerobic) conditions. The capability of anaerobic growth of *S. cerevisiae* can be understood as a consequence of the whole-genome duplication that allowed acquisition of a new physiological property.

## Materials and Methods

### Strains and media

Yeast strains are listed in **Table 3**. Yeast cells were routinely grown at 28°C in a complete YP medium (1% yeast extract, 1% peptone, and 2% glucose), or synthetic minimal medium (0.67% Yeast Nitrogen Base without amino acid, and 2% glucose) supplemented with auxotrophic nutrients. The antibiotic G418 was added to the complete medium when required (200 µg ml^−1^). The media for hypoxic growth were complemented with 30 µg ml^−1^ of ergosterol and 0.2% Tween 80 (polyoxyethylene sorbitan monooleate), and the hypoxic condition was established through 5-minutes air evacuation and 5-minutes nitrogen filling, repeated three times in sealed flasks.

**Table 3:** Yeast strains used in this study.

Yeast strains	Genotype	Source
*K. lactis* strains		
MW270-7B	*MAT* **a** *uraA1-1, leu2, metA1-1*	M. Wésolowski-Louvel, University of Lyon 1
Δklrox1	MW270-7B *klrox1::KanMX*	This work
Δklmot3	MW270-7B *klmot3::KanMX*	This work
*S. cerevisiae* strains		
W303-1B	*MAT* **a** *ade2-1, leu2-3, ura3-1, trp1-1, his3-11, 3-15, can1-100*	R. Rothstein, Columbia University
Δrox1	W303-1B *rox1::KanMX*	This work

### Gene disruption

Deletion of the *S. cerevisiae ROX1* gene was performed by a PCR (polymerase chain reaction)-based one step disruption procedure. A disruption cassette containing a *KanMX* gene was PCR-amplified (primers: 5′-TACTAATACTTCTTCACACAAAAGAACGCAGTTAGACAATCAACAGACATGGAGGCCCAGAATAC-3′ and 5′-ATAGTATAATATAATATAACGGAAAGAAGAAATGGAAAAAAAAAAGTATCGAATCGACAGCAGT-3′), and transformed into the *S. cerevisiae* strain W303-1B. Correct integration was verified by Southern hybridization: the entire open reading frame of *ROX1*, exactly from the initiation codon ATG to the stop codon TGA, was deleted and replaced by the *KanMX* gene to result in a Δ*rox1* mutant.

*K. lactis* Δ*klrox1* and Δ*klmot3* null mutants were constructed by “split-marker recombination” [69]. The DNA fragments corresponding to the upstream and downstream flanking regions of *KlROX1* or *KlMOT3* were amplified by PCR (2 pairs of primers for *KlROX1*: 5′-CGGGATCCGATCTATTCTCATAACTTCGGG-3′, 5′-GGGGTACCTGACAAACCGACAGACTCATAC-3′ and 5′-CGGGATCCGAGAAACTCTGGTTCTAGTCCG-3′, 5′-TACCATTCGGCATATCACAG-3′; for *KlMOT3*: 5′-CGGGATCCGTTCAGCCGTGTGCATCTTC-3′, 5′-GGGGTACCATTCGGACCTATATCAGCATCC-3′ and 5′-CGGGATCCGTGTAGCTAACACCGCGTTGG-3′, 5′-GGGGTACCTGTCTTGCGTTTGGTATTGC-3′), and cloned into pKA and pAN vectors [69] respectively. The resulting plasmids were co-transformed into the *K. lactis* strain MW270-7B. Expected structure of integration was confirmed by Southern hybridization: the sequences from the 7^th^ codon to the 373^rd^ codon of *KlROX1* and from the 15^th^ codon to the 429^th^ codon of *KlMOT3* were deleted and replaced by a *KanMX* selection marker respectively, to give a Δ*klrox1* mutant and a Δ*klmot3* mutant.

### Cloning of the *KlROX1* gene into the expression vector

The open reading frame of *KlROX1* was amplified by PCR (primers: 5′-GCGCGGCCGCATGAGTCTGTCGGTTTGTCATAGAC-3′ and 5′-GCCTGCAGTTTGATTTTGGGGATTGCTCT-3′) from *K. lactis* genomic DNA, and inserted into the *Not*I-*Pst*I site of a 2μ-based multi-copy vector pCM262 [70]. The resulting plasmid pCM262/KlROX1 contained a *KlROX1*-*HA* (a haemoagglutinin epitope) fusion expression cassette.

### Cell-free protein extraction and Western blot

Cells grown to an early stationary phase in minimal medium were harvested, washed and resuspended in a buffer containing 20 mM Tris-HCl, pH 8.0, 50 mM ammonium acetate, 2 mM EDTA and 2 mM phenylmethysulfonyl fluoride (PMSF). One volume of 20% trichloroacetic acid (TCA) was added. The cells were then disrupted with glass beads four times by a vigorous shaking for 1 minute followed by a cooling on ice for 1 minute. The mixture was centrifuged and the protein pellet was washed with acetone and dissolved in gel loading buffer. Proteins were separated in 10% SDS-polyacrylamide gels, transferred to Hybond-C Extra membrane (Amersham), and probed with specific monoclonal antibody 12CA5 for the HA epitope. The reacted protein band was visualized using the ECL chemiluminescence detection system (Amersham).

### RNA extraction and Northern hybridization

Total RNA was isolated as described [71], fractionated on an agarose/formaldehyde denaturing gel, and immobilized onto a Hybond-N^+^ membrane (Amersham). Hybridization was performed at 65°C in a buffer containing 7% SDS, 0.5 M sodium phosphate buffer, pH 7.2, and 10 mM EDTA. Probes were synthesized by PCR (oligonucleotides for *HEM13* amplification: 5′-GATCCAAGGAATCTTCCAAT-3′ and 5′-TAACCATGAAGCATGTTCAG-3′; for *KlHEM13*: 5′-TCCATTCGACTCACCAACTG-3′ and 5′-TTCAAACCCATTCAACAGGG-3′; for *KlHYP2*(*ANB1*): 5′-CCAAACGCATTAAACAAATCA-3′ and 5′-CTTCTTTCCATTTATCCAGGG-3′, and for *KlCOX5A*(*5B*): 5′-CCACTTGCAATTATCGTCTGA-3′ and 5′-AGAAGAGAGGGAGAAAATGCA-3′), and labelled with ^32^P using the ‘Ready to Go DNA Labelling Kit’ (Pharmacia).

### Comparative genome analysis in yeasts

BLAST search was performed using tools implemented at the database websites of the yeast species (S. cerevisiae, Saccharomyces paradoxus, Saccharomyces mikatae, Saccharomyces bayanus, Saccharomyces castellii, Saccharomyces kluyveri, and Kluyveromyces waltii: http://db.yeastgenome.org/cgi-bin/FUNGI/showAlign or/and http://seq.yeastgenome.org/cgi-bin/blast-fungal.pl?name; Candida glabrata, K. lactis, Debaryomyces hansenii and Yarrowia lipolytica: http://cbi.labri.fr/Genolevures/blast/index.php; Candida albicans: http://www.candidagenome.org/cgi-bin/nph-blast; Schizosaccharomyces. pombe: http://www.genedb.org/genedb/pombe/blast.jsp).

Synteny comparison was carried out manually or viewed at http://db.yeastgenome.org/cgi-bin/FUNGI/FungiMap?locus (*S. cerevisiae*, *S. paradoxus*, *S. mikatae* and *S. bayanus*) and http://wolfe.gen.tcd.ie/cgi/browser/ygob.pl?gene (*S. cerevisiae*, *S. bayanus*, *C. glabrata*, *S. castellii*, *K. waltii*, *S. kluyveri*, and *K. lactis*).

### Mathematical calculation

#### Cumulative binomial distribution

To determine the significance of the difference of the fraction of a certain property between two samples (*i.e.*, datasets), we used the formula below to calculate *P*-values:
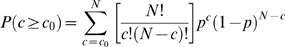
where *N* is the total number of genes in the testing sample, *c* is the number of genes with a specific property and 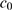 the number of observed genes with this property, and *p* is the probability of finding a gene with the same property randomly (picking from the entire genome) or in the control sample.

#### Stochastic model of the site YYYATTGTTCTC

According to a previous report about the DNA sequence requirements of the consensus Rox1p-binding site Y_1_Y_2_Y_3_A_4_T_5_T_6_G_7_T_8_T_9_C_10_T_11_C_12_ [53], the sequence T_5_T_6_G_7_T_8_ is absolutely required for the affinity of Rox1p binding; A_4_ can be relatively tolerated to substitution by T, T_9_ by C or A and Y_3_ by A; A_4_ and T_9_ can not be permitted to change at the same time. With these limitations of mismatches allowed, we searched for the possible binding sites in the upstream sequences (1 kb) of both the thirty-seven Rox1p-target genes of *S. cerevisiae* and their forty-one orthologs of *K. lactis* (see Results). And we also calculated the stochastic probabilities that the site YYYATTGTTCTC appears in thirty-seven and forty-one 1-kb DNA sequences as described below:

Lemma 1 [72]: Let 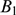, 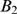 …, 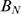 denote the events in some probability space. Then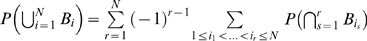, and 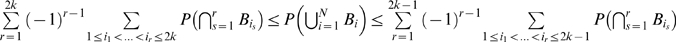 holds for all 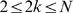, where the sum 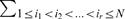 is taken over all nonnegative integers with 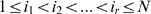.

Let 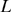 denote the string “
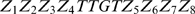
”, where 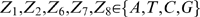, 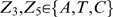, and 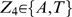. Define 
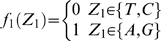
, 
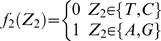
, 
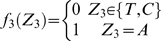
, 
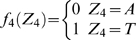
, 
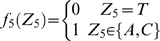
, 
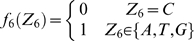
, 
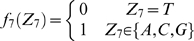
 and 
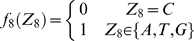
. Let 
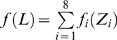
 and 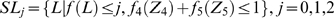 (prefect match, 1 mismatch allowed, 2 mismatches allowed).

DNA is double-stranded. For complementary strand, let 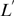 denote the string “
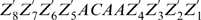
”, where 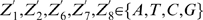, 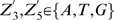 and 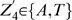. Define 
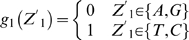
, 
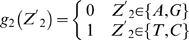
, 
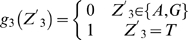
, 
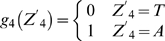
, 
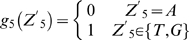
, 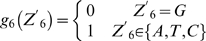, 
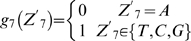
 and 
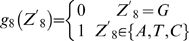
. Let 
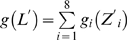
. Define 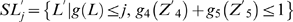 (prefect match, 1 mismatch allowed, 2 mismatches allowed).

Let 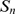 be the probability space consisting of all the character strings of length 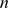 on set 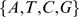 with equality probability. Now we can choose a string 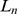 from 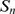 at random. For 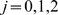, let 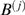 (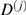) be the event that 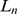 contains substring 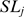 (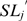) and 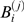 (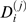) be the event that the substring of 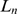 consisting of 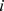 st character to 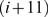 st character is 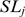 (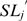). Then 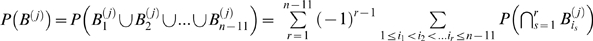 and 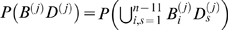.

By Lemma 1, we can have that 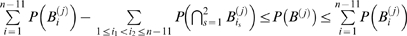 and 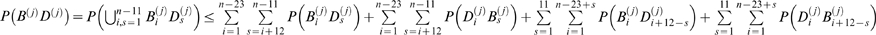. By symmetry, 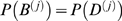. Since 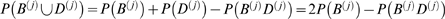, we can know that 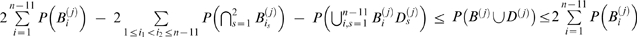, in which 
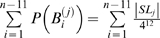
 and 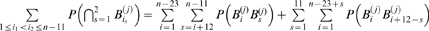.

Let 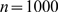. If 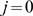, 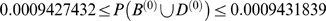, that is 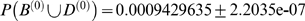; If 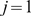, 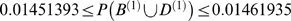, that is 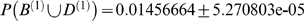; If 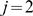, 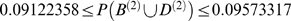, that is, 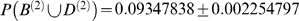.

Now we can choose 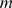 strings of length 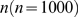 from 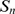. For 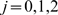, let 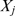 be the number of the strings that contains substring 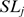 or 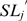. Let 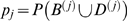, then 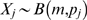, that is 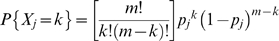, where 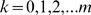.

## Supporting Information

Table S1 List of oxygen-responding genes in the ohnologs of S. cerevisiae.
(0.48 MB XLS)

Table S2 Significance of distribution of oxygen-responding genes in the ohnologs of S. cerevisiae.
(0.10 MB DOC)

Table S3 Number of YYYATTGTTCTC site in the upstream sequences of Rox1p-mediated genes in S. cerevisiae and of the orthologs in K. lactis.
(0.14 MB DOC)
