# Supplementary material for: Gene Responses to Oxygen Availability in Kluyveromyces lactis: an Insight on the Evolution of the Oxygen-Responding System in Yeast
Source: PLoS One. 2009 Oct 26;4(10):e7561. doi: 10.1371/journal.pone.0007561 (PMC2763219; doi:10.1371/journal.pone.0007561)
Supplement: Table S2 — Significance of distribution of oxygen-responding genes in the ohnologs of S. cerevisiae. (0.10 MB DOC) [file pone.0007561.s002.doc]

**Table S2. Significance of distribution of oxygen-responding genes in the ohnologs of *S. cerevisiae***

A. ohnolog versus whole genome

| O_2_ regulation | Whole genome (6020 genes) | | | | Ohnolog (1108 genes) | | | | *P*-value |
| --- | --- | --- | --- | --- | --- | --- | --- | --- | --- |
|  | O_2_-responding | | Percentage (%) | | O_2_-responding | | Percentage (%) | |  |
|  |  | |  | |  | |  | |  |
| 1.5 fold |  | |  | |  | |  | |  |
| Up | 642 |  | 10.66 |  | 175 |  | 15.79 |  | 1.14e-07 |
| Down | 703 |  | 11.68 |  | 165 |  | 14.89 |  | 7.36e-04 |
| Total | 1345 |  | 22.34 |  | 340 |  | 30.69 |  | 8.26e-11 |
|  |  |  |  |  |  |  |  |  |  |
| 2 fold |  |  |  |  |  |  |  |  |  |
| Up | 330 |  | 5.48 |  | 92 |  | 8.30 |  | 6.96e-05 |
| Down | 390 |  | 6.48 |  | 115 |  | 10.38 |  | 6.46e-07 |
| Total | 720 |  | 11.96 |  | 207 |  | 18.68 |  | 7.13e-11 |
|  |  |  |  |  |  |  |  |  |  |
| 3 fold |  |  |  |  |  |  |  |  |  |
| Up | 148 |  | 2.46 |  | 39 |  | 3.52 |  | 1.84e-02 |
| Down | 225 |  | 3.74 |  | 63 |  | 5.69 |  | 8.56e-04 |
| Total | 373 |  | 6.20 |  | 102 |  | 9.21 |  | 5.80e-05 |

B. ohnolog versus non-ohnolog

| O_2_ regulation | Non-ohnolog (4912 genes) | | | | Ohnolog (1108 genes) | | | | *P*-value |
| --- | --- | --- | --- | --- | --- | --- | --- | --- | --- |
|  | O_2_-responding | | Percentage (%) | | O_2_-responding | | Percentage (%) | |  |
|  |  | |  | |  | |  | |  |
| 1.5 fold |  | |  | |  | |  | |  |
| Up | 467 |  | 9.51 |  | 175 |  | 15.79 |  | 2.92e-11 |
| Down | 538 |  | 10.95 |  | 165 |  | 14.89 |  | 3.53e-05 |
| Total | 1005 |  | 20.46 |  | 340 |  | 30.69 |  | 6.84e-16 |
|  |  |  |  |  |  |  |  |  |  |
| 2 fold |  |  |  |  |  |  |  |  |  |
| Up | 238 |  | 4.85 |  | 92 |  | 8.30 |  | 6.24e-07 |
| Down | 275 |  | 5.60 |  | 115 |  | 10.38 |  | 1.82e-11 |
| Total | 513 |  | 10.44 |  | 207 |  | 18.68 |  | 1.86e-16 |
|  |  |  |  |  |  |  |  |  |  |
| 3 fold |  |  |  |  |  |  |  |  |  |
| Up | 109 |  | 2.22 |  | 39 |  | 3.52 |  | 3.98e-03 |
| Down | 162 |  | 3.30 |  | 63 |  | 5.69 |  | 3.18e-05 |
| Total | 271 |  | 5.52 |  | 102 |  | 9.21 |  | 4.96e-07 |
