# Supplementary material for: Gene Responses to Oxygen Availability in Kluyveromyces lactis: an Insight on the Evolution of the Oxygen-Responding System in Yeast
Source: PLoS One. 2009 Oct 26;4(10):e7561. doi: 10.1371/journal.pone.0007561 (PMC2763219; doi:10.1371/journal.pone.0007561)
Supplement: Table S3 — Number of YYYATTGTTCTC site in the upstream sequences of Rox1p-mediated genes in S. cerevisiae and of the orthologs in K. lactis. (0.14 MB DOC) [file pone.0007561.s003.doc]

**Table S3. Number of YYYATTGTTCTC site in the upstream sequences of Rox1p-mediated genes in *S. cerevisiae* and of the orthologs in *K. lactis***

| ***S. cerevisiae*** | | | | | | ***K. lactis*** | | | | | |
| --- | --- | --- | --- | --- | --- | --- | --- | --- | --- | --- | --- |
| Gene/ORF | Mismatches allowed | | | | | Gene/ORF | Mismatches allowed | | | | |
|  | 4 | 3 | 2 | 1 | 0 |  | 4 | 3 | 2 | 1 | 0 |
| *AAC3 ^a^* | 3 | 2 | 1 | 0 | 0 | KLLA0E12353g | 2 | 1 | 0 | 0 | 0 |
| *FET4* | 3 | 3 | 1 | 0 | 0 | KLLA0E14564g | 1 | 0 | 0 | 0 | 0 |
| *ANB1 ^a^* | 5 | 4 | 4 | 3 | 2 | KLLA0E22286g | 3 | 2 | 0 | 0 | 0 |
| *HEM13 ^b^* | 8 | 3 | 1 | 1 | 1 | KLLA0F18546g | 3 | 2 | 1 | 1 | 0 |
| *COX5B ^a^* | 5 | 2 | 0 | 0 | 0 | KLLA0F03641g | 3 | 1 | 0 | 0 | 0 |
| *OYE2* | 3 | 2 | 1 | 1 | 0 | KLLA0A09075g | 3 | 0 | 0 | 0 | 0 |
| *LAC1 ^a^* | 5 | 4 | 2 | 0 | 0 | KLLA0B13497g | 2 | 1 | 0 | 0 | 0 |
|  |  |  |  |  |  | KLLA0E08141g | 5 | 4 | 0 | 0 | 0 |
| *YMR252C ^b^* | 5 | 5 | 3 | 1 | 1 | KLLA0B08778g | 1 | 1 | 0 | 0 | 0 |
|  |  |  |  |  |  | KLLA0F22792g | 3 | 2 | 1 | 0 | 0 |
| *FRDS1 ^a^* | 8 | 3 | 2 | 1 | 0 | KLLA0F16753g | 3 | 0 | 0 | 0 | 0 |
| *SUR2 ^b^* | 2 | 1 | 0 | 0 | 0 | KLLA0C10406g | 0 | 0 | 0 | 0 | 0 |
| *SML1 ^a^* | 3 | 3 | 1 | 1 | 0 | KLLA0B05423g | 4 | 2 | 1 | 0 | 0 |
| *YNR014W ^a^* | 2 | 2 | 1 | 0 | 0 | KLLA0F06138g | 1 | 0 | 0 | 0 | 0 |
| *FRT2 ^a^* | 8 | 5 | 4 | 1 | 0 | KLLA0A03938g | 1 | 1 | 0 | 0 | 0 |
| *DIP5 ^b^* | 2 | 2 | 2 | 0 | 0 | KLLA0E16335g | 1 | 0 | 0 | 0 | 0 |
| *ISU2 ^a^* | 2 | 2 | 0 | 0 | 0 | KLLA0D07161g | 3 | 2 | 1 | 0 | 0 |
| *EUG1 ^a^* | 3 | 2 | 0 | 0 | 0 | KLLA0C01111g | 0 | 0 | 0 | 0 | 0 |
| *HEM14 ^b^* | 3 | 2 | 1 | 1 | 1 | KLLA0B11616g | 2 | 1 | 0 | 0 | 0 |
| *PIS1 ^b^* | 1 | 1 | 1 | 0 | 0 | KLLA0D15037g | 1 | 1 | 0 | 0 | 0 |
| *ERG26 ^b^* | 6 | 3 | 2 | 1 | 1 | KLLA0C18513g | 2 | 1 | 0 | 0 | 0 |
| *YGR066C* | 7 | 0 | 0 | 0 | 0 | KLLA0B09592g | 2 | 0 | 0 | 0 | 0 |
| *SPI1 ^a^* | 3 | 1 | 1 | 0 | 0 | KLLA0E01057g | 6 | 4 | 0 | 0 | 0 |
| *SCM4 ^a^* | 3 | 2 | 1 | 0 | 0 | KLLA0A02695g | 2 | 0 | 0 | 0 | 0 |
| *CWP1 ^b^* | 3 | 3 | 2 | 0 | 0 | KLLA0B06347g | 4 | 1 | 0 | 0 | 0 |
| *RTS3 ^b^* | 2 | 1 | 1 | 0 | 0 | KLLA0C11539g | 3 | 2 | 1 | 0 | 0 |
| *CYB5 ^b^* | 4 | 2 | 1 | 0 | 0 | KLLA0F23672g | 2 | 1 | 1 | 0 | 0 |
| *FIT2* | 4 | 2 | 0 | 0 | 0 | KLLA0C05016g | 0 | 0 | 0 | 0 | 0 |
|  |  |  |  |  |  | KLLA0A04367g | 2 | 2 | 0 | 0 | 0 |
| *RTA1 ^a^* | 2 | 2 | 1 | 1 | 0 | KLLA0F00264g | 3 | 1 | 0 | 0 | 0 |
| *MCH5 ^b^* | 2 | 2 | 2 | 0 | 0 | KLLA0F08525g | 3 | 1 | 0 | 0 | 0 |
| *CYC7 ^a^* | 2 | 0 | 0 | 0 | 0 | KLLA0F16929g | 0 | 0 | 0 | 0 | 0 |
| *GLC7* | 1 | 0 | 0 | 0 | 0 | KLLA0F12496g | 3 | 2 | 1 | 0 | 0 |
| *FIT3* | 1 | 1 | 0 | 0 | 0 | KLLA0C04928g | 5 | 2 | 1 | 1 | 0 |
| *ERG11 ^b^* | 5 | 4 | 1 | 0 | 0 | KLLA0E03553g | 0 | 0 | 0 | 0 | 0 |
| *NCP1* | 3 | 3 | 3 | 1 | 0 | KLLA0D08712g | 3 | 2 | 1 | 0 | 0 |
| *OLE1 ^b^* | 3 | 3 | 1 | 1 | 0 | KLLA0C05566g | 2 | 0 | 0 | 0 | 0 |
|  |  |  |  |  |  | KLLA0C10692g | 1 | 0 | 0 | 0 | 0 |
| *HMG2 ^a^* | 3 | 3 | 1 | 1 | 1 | KLLA0B04642g | 7 | 4 | 3 | 0 | 0 |
| *SUT1 ^a^* | 4 | 0 | 0 | 0 | 0 | KLLA0A03091g | 3 | 1 | 0 | 0 | 0 |
| *ATF1* | 3 | 1 | 1 | 0 | 0 | KLLA0F15202g | 3 | 1 | 1 | 0 | 0 |
| Total | 132 | 81 | 43 | 15 | 7 | Total | 98 | 46 | 13 | 2 | 0 |

*^a^* The gene has an ohnolog. *^b^* The gene is single but located within the duplication block

Searching for putative Rox1p binding site YYYATTGTTCTC (in two orientations) was carried out over a 1-kb region upstream of each gene by using the program at <http://rulai.cshl.edu/SCPD/searchconsensus.html>.
